# Recurrent Pneumothorax in *PIEZO2*‐Related Arthrogryposis: Implications for the Mechanosensory Function of *PIEZO2* in the Respiratory System

**DOI:** 10.1002/cga.70045

**Published:** 2026-02-16

**Authors:** Daisuke Nakato, Ikumi Ono, Kumiko Misu, Fuyuki Miya, Kenjiro Kosaki

**Affiliations:** ^1^ Center for Medical Genetics, Keio University School of Medicine Tokyo Japan

## 1 Introduction

Arthrogryposis is a clinically and genetically heterogeneous disorder characterized by congenital multiple joint contractures. More than 400 causative genes have been identified to date. Arthrogryposis is relatively common, occurring in approximately 1 in 3000 individuals, and most cases are clinically non‐progressive [1]. Among these causative genes, pathogenic variants in *PIEZO2* have been reported as a genetic cause of arthrogryposis [2]. *PIEZO2* encodes a mechanosensitive ion channel that plays a critical role in proprioception and skeletal development, and dysfunction of this pathway has been implicated in skeletal manifestations such as scoliosis [3].

The clinical spectrum is broad, extending to syndromic presentations such as Marden–Walker phenotype with Dandy–Walker malformation, in addition to other skeletal and developmental manifestations [4]. *PIEZO2*‐related arthrogryposis may involve thoracic deformity and scoliosis that restrict chest wall motion and lead to a restrictive ventilatory pattern. Individuals with *PIEZO2* dysfunction have been reported to show reduced lung volumes and impaired lung expansion, consistent with restrictive pulmonary changes due to limited thoracic mobility [2, 5]. In addition, scoliosis and axial skeletal manifestations commonly observed in these patients may further decrease thoracic compliance and exacerbate respiratory restriction [3].

Mouse models of *PIEZO2* deficiency have been developed, and many of their phenotypic features recapitulate those observed in human patients with PIEZO2 variants [3, 5]. These models exhibit abnormalities of the joints, like those described in affected individuals, supporting the relevance of *PIEZO2* dysfunction to the skeletal manifestations of the disorder. Interestingly, conditional knockout of PIEZO2 has been shown to result in excessive expansion of the pulmonary alveoli, a unique feature that is not observed in other forms of arthrogryposis [5]. It remains unknown whether patients exhibit clinical features associated with alveolar changes.

Here, we report a patient with a pathogenic *PIEZO2* variant who presented with a phenotype consistent with Marden–Walker syndrome with Dandy–Walker malformation and experienced recurrent spontaneous pneumothorax associated with bullae.

## 2 Clinical Presentation

The patient was born by cesarean section at 39 + 3 weeks of gestation because of breech presentation. His birth weight was 2340 g (−2.1 SD) and length was 40.5 cm (−4.1 SD). At birth, he was admitted to the neonatal intensive care unit with respiratory compromise and multiple congenital anomalies.

**FIGURE 1:**
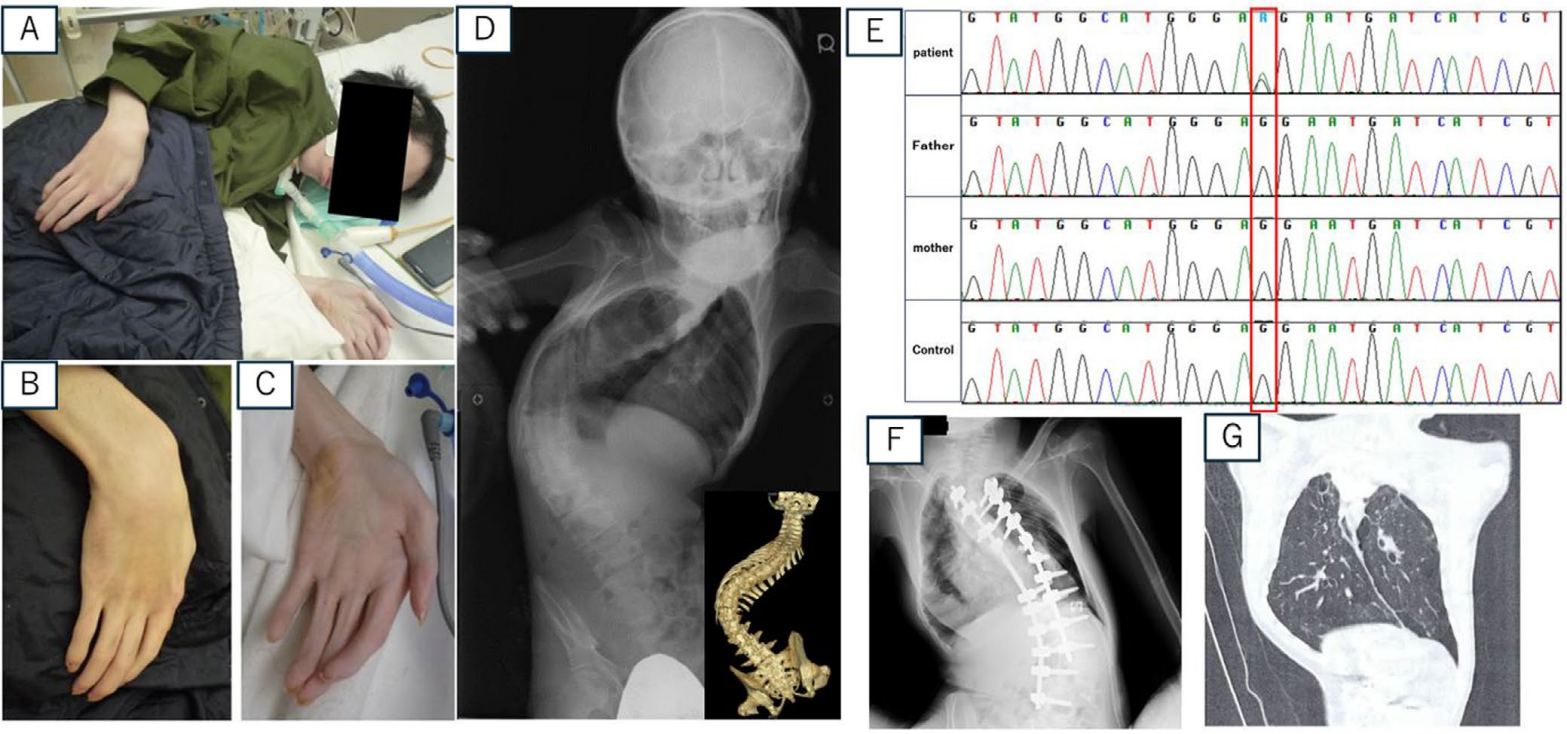
Clinical and radiologic findings of the patient and Genetic analysis of *PIEZO2*. (A–C) Distal arthrogryposis with contractures of the hands and feet. (D) Progressive scoliosis observed during adolescence. (E) Whole‐exome sequencing identified a heterozygous de novo missense variant in *PIEZO2* (NM_022068.4: c.7216G>A, p. Gly2406Arg) in exon 46. The variant was absent in both parents and was classified as likely pathogenic according to ACMG/AMP criteria. (F) Chest radiograph showing bilateral spontaneous pneumothorax. (G) Chest computed tomography demonstrates multiple pre‐existing pulmonary bullae in both lungs.

During infancy and childhood, he showed distal arthrogryposis (Figure 1A–C) and mild hearing loss. Development was mildly delayed, with rolling at 12 months, first words at 18 months, and independent standing at 3 years. Brain MRI revealed Dandy–Walker malformation. His scoliosis progressed during adolescence (Figure 1D), and corrective surgery was performed at age 14. Because there was no family history of joint contractures or scoliosis, genetic etiology was suspected and genetic testing was undertaken. Figure 1E provides an overview of the genetic findings.

At age 21, he presented with acute dyspnea while showering and was diagnosed with bilateral spontaneous pneumothorax (Figure 1F). Chest CT revealed numerous pre‐existing bullae in both lungs (Figure 1G). At age 22, he developed a second pneumothorax complicated by tension pneumothorax, resulting in cardiopulmonary arrest; he underwent resuscitation but subsequently developed Lance–Adams syndrome secondary to hypoxic–ischemic encephalopathy and required anticonvulsant therapy. A third pneumothorax occurred at age 23. Owing to severe scoliosis, arthrogryposis, and difficult airway management, he is currently managed at home with a tracheostomy and careful respiratory support.

## 3 Discussion

Here we report a patient with *PIEZO2*‐related arthrogryposis who developed recurrent spontaneous pneumothorax with multiple bullae. Although respiratory involvement has been noted in individuals with PIEZO2 dysfunction, pneumothorax has not previously been documented in this setting [6]. The patient experienced multiple recurrent pneumothorax episodes, one of which resulted in cardiopulmonary arrest, underscoring the clinical seriousness of this complication.

The mechanism of recurrent pneumothorax in this patient remains unknown; however, there is a striking phenotypic resemblance between the patient and a *PIEZO2*‐deficient loss‐of‐function mouse model [5]. The presence of multiple bullae in this patient may reflect a combination of pulmonary overinflation related to *PIEZO2* dysfunction and impaired tidal volume regulation, which could increase mechanical stress on the lung and predispose to pneumothorax. However, the absence of longitudinal imaging before pneumothorax onset precludes determination of whether the bullae were a primary manifestation of *PIEZO2*‐related pulmonary pathology or incidental findings.

Previous reports have shown that heterozygous missense mutations in *PIEZO2* associated with distal arthrogryposis syndromes lead to increased channel activity, whereas biallelic loss‐of‐function mutations abolish mechanotransduction. The *PIEZO2* variant identified in our patient, p.Gly2406Arg, is a heterozygous missense mutation located in the C‐terminal region and, to our knowledge, has not been reported in previous studies. Based on these previous findings, p.Gly2406Arg is likely to alter PIEZO2 channel gating or kinetics; however, functional validation will be required to confirm the precise effect of this variant.

This finding provides an important clue. Nonomura et al. demonstrated that *PIEZO2*‐deficient mice exhibit overinflation of the lungs and speculated that disruption of the Hering–Breuer reflex—which normally limits lung inflation via a vagal feedback pathway—is absent in these mouse models [5]. In these mice, loss of nodose ganglion neurons essential for this reflex was observed. A similar impairment may also be present in the patient described here, and further research is warranted.

In summary, our findings broaden the respiratory phenotype of PIEZO2‐related disorders and indicate that pneumothorax, including recurrent events, should be recognized as a potential complication. These observations further suggest that pneumothorax may represent part of the clinical spectrum, underscoring the need for respiratory surveillance and early intervention.

## Funding

This work was supported by the Japan Agency for Medical Research and Development (AMED) under grant numbers JP25ek0109760.

## Ethics Statement

This study was conducted in accordance with the Declaration of Helsinki and was approved by the Ethics Committee of Keio University School of Medicine.

## Consent

Written informed consent for publication was obtained from the patient, and the signed consent form is on file in the medical record. The authors confirm that all identifiable information has been anonymized.

## Conflicts of Interest

The authors declare no conflicts of interest.

## Data Availability

The data that support the findings of this study are available from the corresponding author upon reasonable request.
